# Performance of magnetic-resonance imaging radiomics in prediction of response after neoadjuvant chemotherapy in head and neck squamous cell carcinoma: A systematic review and meta-analysis

**DOI:** 10.1016/j.ejro.2026.100786

**Published:** 2026-06-22

**Authors:** Saeed Mohammadzadeh, Fatemeh Mahdavi Sabet, Iman Kiani, Seyed Amir Mohammad Seyed Rahmani, Sajad Mohammadzadeh, Farzad Fayedeh, Houman Sotoudeh

**Affiliations:** a Advanced Diagnostic and Interventional Radiology Research Center (ADIR), Tehran University of Medical Sciences, Imam Khomeini Hospital, Tehran, Iran; b School of Medicine, Tehran University of Medical Sciences, Tehran, Iran; c Faculty of Medicine, Tehran Medical Sciences Branch, Islamic Azad University, Tehran, Iran; d School of Medicine, Golestan University of Medical Sciences, Gorgan, Iran; e School of Medicine, Birjand University of Medical Sciences, Birjand, Iran; f Associate Professor of Radiology Neuroradiology Section UT Southwestern Medical Center, Dallas, TX, USA

**Keywords:** Radiomics, Head and neck squamous cell carcinoma, MRI, response assessment

## Abstract

**Objective:**

To evaluate performance of MRI-based radiomics for evaluating response to neoadjuvant chemotherapy (NACT) in head and neck squamous cell carcinoma (HNSCC) patients.

**Method:**

We performed a comprehensive search across four databases until July, 2025 to include studies evaluating diagnostic performance of MRI radiomics in response assessment. Methodological quality of studies was assessed using METhodological RadiomICs Score (METRICS). Using a random-effects bivariate model diagnostic values of area under the curve (AUC), sensitivity, and specificity were calculated. Meta-regression and subgroup analyses were used to explore the source of heterogeneity.

**Results:**

Twenty-one studies were included. The overall predictive performance of MRI-radiomics in the validation set was good, with a summary AUC of 0.84 (95% CI: 0.81–0.87) in differentiation of complete or partial response (CR/PR) and non-responders as those with stable or progressive disease (SD/PD). High heterogeneity was observed, but there was no evidence of publication bias (Deeks’ test p = 0.20). Subgroup analysis resolved the heterogeneity and showed similar performance of model across different therapeutic regimens, study designs, study qualities, and MRI magnitudes of strength. For classifying only CR as responders and PR + SD + PD as non-responders, MRI-radiomics achieved an AUC of 0.83 (95% CI: 0.79–0.86) with low heterogeneity.

**Conclusion:**

MRI radiomics demonstrated high predictive value in evaluating NACT response for HNSCC patients, showing consistent performance across different clinical scenarios. However, the current evidence base is largely retrospective, geographically limited, and predominantly derived from nasopharyngeal cancer cohorts; therefore, broader external validation and prospective multicenter designs are required before routine clinical implementation.

## 1 Introduction

Head and neck squamous cell carcinoma (HNSCC) is regarded as the seventh most prevalent malignancy worldwide, affecting several anatomic locations in the upper aerodigestive tract [1]. There have been reports of an increase in occurrence, which has been linked to human papilloma virus (HPV) infection [2]. Over two-thirds of newly diagnosed HNSCC cases are found at a locally advanced stage, and multimodality treatment involving surgery, chemotherapy, and/or radiotherapy has led to better disease management and survival [3], [4]. Tumor heterogeneity in HNSCC limits the effectiveness of standardized treatment approaches. Within HNSCC, nasopharyngeal carcinoma warrants specific distinction as a unique subtype comprising ∼3–5% of global cases but up to 30% in endemic regions like East Asia, primarily linked to Epstein-Barr virus (EBV) rather than tobacco, alcohol, or HPV (which predominates in oropharyngeal sites). nasopharyngeal carcinoma's treatment paradigm also differs, emphasizing neoadjuvant chemotherapy (NACT) followed by chemoradiotherapy over surgery used in other sites, reflecting its radiosensitivity and biology [5]. Personalized treatment strategies are therefore essential to improve survival and reduce adverse effects [6]. NACT has shown promising results in reduction of distant metastases, especially in laryngeal and hypopharyngeal carcinomas. It has also been shown to significantly increase the survival of patients with advanced inoperable HNSCCs [7]. Furthermore, NACT has shown to benefit organ preservation, and longer progression of the disease [8]. In patients with advanced HNSCC, obtaining a histologically full response prior to surgery and following NACT may enhance locoregional control and overall survival [9], [10].

Advancements in medical imaging have resulted in enhancement of imaging techniques, contrast agents, standardized protocols, and analytical methods like radiomics [11]. Radiomics involves the extraction of numerous quantitative features using automated or semi-automated software. It is based on the assumption that extractable data from medical images can yield further insights into tumor characteristics, which can subsequently be applied to patient treatment [11], [12], [13]. In fact, intra-tumor heterogeneity has been noted to significantly influence clinical predictions, such as treatment response, survival rates, disease advancement, and so forth, making it an essential component for precision oncology and associated studies [14], [15], [16]. Several studies have been conducted in the use of radiomics for detection and also prognosis of HNSCC [17], [18], [19], [20]. While CT offers standardized performance across scanners, acquisition parameters, reconstruction algorithms, and noise still impact feature reliability. MRI radiomics holds greater potential than CT, as it can capture diverse voxel properties through multi-parametric sequences, including diffusion-weighted imaging (DWI), apparent diffusion coefficient (ADC) mapping, dynamic contrast-enhanced studies, and MR perfusion (MRP) [21]. These developments highlight the potential of radiomics, to serve as a non-invasive tool for predicting treatment response and guiding personalized management in HNSCC.

Several preliminary studies have explored the potential of radiomics in assessing treatment response in HNSCC [22], [23], [24]. However, to our knowledge, there has been no comprehensive systematic review and meta-analysis evaluating its performance for predicting response after NACT. Addressing this gap is crucial, as artificial intelligence-driven assessment of complete response (CR) could help avoid unnecessary surgery, facilitate organ preservation, and enable more precise treatment strategies. Therefore, the aim of this study is to systematically review and quantitatively synthesize the available evidence on the predictive performance of MRI radiomics in predicting treatment response and to explore its potential for clinical translation in HNSCC patients.

## 2 Methods

We designed our methodological approach based on the detailed recommendations in the Cochrane Handbook for Systematic Reviews of Diagnostic Test Accuracy. To maintain consistency in reporting, we followed the Preferred Reporting Items for Systematic Reviews and Meta-Analyses of Diagnostic Test Accuracy (PRISMA-DTA) guidelines [25]. We also relied on the PRISMA-Search (PRISMA-S) framework [26], which standardizes search strategies and supports a systematic and exhaustive identification of relevant studies. The study protocol was pre-registered at the PROSPERO registry (ID: *anonymized*), to ensure transparency and reproducibility.

### 2.1 Search strategy

Two reviewers independently designed search strategies using MeSH and EMTREE terms. Then the strategies were compared, refined, and consolidated into a unified search strategy for each database **(**Supplementary material**)**. Any disagreements were resolved through discussion with a third reviewer. A comprehensive search was carried out on July 21, 2025, across four databases: PubMed, Embase, Web of Science, and Scopus. The search keywords included radiomics, MRI, neoadjuvant chemotherapy, pathological complete response, head and neck cancer and other relevant free words with no language restrictions were applied. The retrieved studies were imported into Rayyan and duplicate records were removed.

### 2.2 Eligibility criteria and study selection

We included observational studies, with cross-sectional, case-control, and cohort designs that evaluated treatment response in HNSCC patients who received NACT (with or without radiotherapy) and underwent MRI examination. Eligible studies were required to assess treatment response using MRI-based radiomics, with the Response Evaluation Criteria in Solid Tumors (RECIST) guidelines serving as the commonly used clinical reference standard. Exclusion criteria were as follows; 1) Case reports, case series, editorials, comments, conference abstracts, review articles, and grey literature published without peer-review process. 2) Studies that utilized clinical data, tumor size measurements, or other self-defined criteria instead of RECIST as the reference standard for response evaluation. 3) Studies that did not report sufficient quantitative data to allow extraction of 2 * 2 contingency Table 4) Studies that evaluated the performance of MRI radiomics solely for predicting overall survival or progression-free survival in HNSCC patients.

Two reviewers independently evaluated the retrieved records for eligibility. Any discrepancies regarding inclusion were resolved by consensus, with a third reviewer justifying unresolved disagreements. Moreover, reference lists of relevant articles were reviewed to ensure the inclusion of all potentially eligible studies. If several studies used the same or a portion of patients, the study with the larger and more comprehensive population was kept.

### 2.3 Quality assessment

In order to ensure the reliability and accuracy of our findings, we assessed each included study independently for methodological quality by two reviewers using the METhodological RadiomICs Score (METRICS) [27]. METRICS is endorsed by the European Society of Medical Imaging Informatics (EuSoMII) to provide a transparent and standardized approach for assessing and enhancing the quality of radiomics and machine learning (ML) researches. This tool quantifies study quality by a score from 0 to 100 based on the 30 pre-defined items in following domains: (1) image protocol standardization, (2) feature extraction robustness, (3) statistical analysis validity, (4) clinical relevance, (5) reliability reporting, and (6) study design.

### 2.4 Data extraction

Two researchers independently extracted all pertinent data from the included studies. They documented their findings in individual Excel files and then compared the entries to detect discrepancies. Unresolved conflicting entries have been delivered to a third investigator for a conclusive determination. The extracted data comprised the author, year, country, study design (single center vs multicenter and prospective vs. retrospective), age, gender (prevalence of males), tumor type and grade, patient count, treatment regimen, MRI field strength and sequence, and definition of treatment response. MRI-based radiomics 2 × 2 contingency table data for assessing treatment status were taken from both training and validation sets. TP indicated accurate radiomic identification of treatment response; FP denoted erroneous radiomic prediction of response in non-responder tumors; FN referred to unrecognized responder tumors; TN was considered valid classification of non-responder tumors by MRI radiomics.

### 2.5 Data synthesis

Statistical analysis was conducted using STATA version 18.0. We implemented a random-effects bivariate pooling model and conducted a publication bias assessment for minor study effects. A sensitivity analysis was conducted to identify outlier studies that may have significantly impacted the overall results. Diagnostic metrics, including the area under the curve (AUC), sensitivity, specificity, positive likelihood ratio (PLR), negative likelihood ratio (NLR), and diagnostic odds ratio (DOR), were utilized for the training and validation datasets. Additionally, receiver operating characteristic (ROC) curves were produced. If studies provided 2 × 2 contingency table data for each radiomics model, we selected the model recommended by the authors or, if unavailable, the best-fitted model to our study question. Included studies were stratified into two distinct groups based on their definition of treatment response according to RECIST criteria. The first group defined responders as patients exhibiting a complete or partial response (CR/PR) and non-responders as those with stable or progressive disease (SD/PD). The second group defined response more specifically, classifying only complete responders (CR) as responders, while identifying partial response, stable disease, and progressive disease (PR/SD/PD) as non-responders. A separate meta-analysis was conducted for each group to assess the predictive efficacy of radiomics according to each response definition. Grading of Recommendations, Assessment, Development, and Evaluations (GRADE) tool was employed to evaluate the certainty of evidence in each result subheading. An I2 value of ≥ 50% signifies high statistical heterogeneity, whereas a p-value < 0.05 qualifies as statistically significant.

## 3 Results

### 3.1 Literature search and study selection

**Fig. 1:**
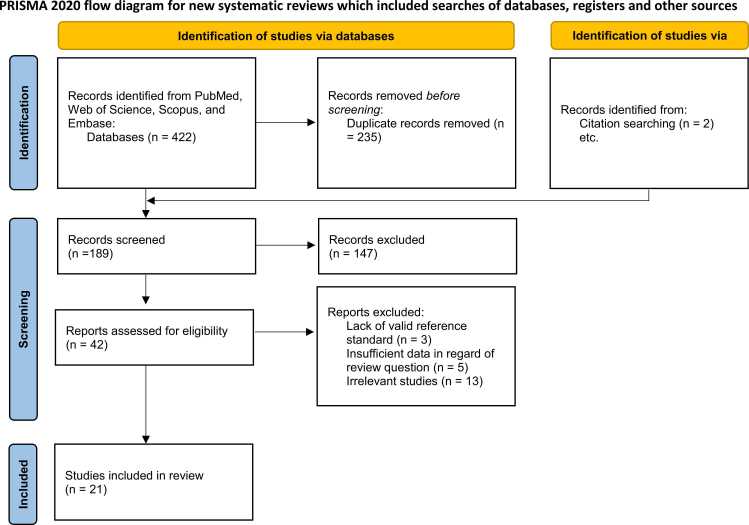
PRISMA flow diagram of the selection process.

The initial database search yielded 422 articles, with 2 additional records identified through citation searching. After removing 235 duplicate records, 189 articles underwent title and abstract screening, of which 147 studies were excluded as they did not meet the eligibility criteria. The full texts of the remaining 42 articles were assessed, resulting in the final inclusion of 21 studies for systematic review and meta-analysis. The PRISMA flow diagram detailing the study selection process is presented in Fig. 1.

### 3.2 Characteristics of included studies

A total of 21 studies comprising 4533 patients were included in the analysis [23], [28], [29], [30], [31], [32], [33], [34], [35], [36], [37], [38], [39], [40], [41], [42], [43], [44], [45], [46], [47]. The baseline characteristics of the included studies are presented in Table 1. The majority of studies were conducted in China (20/21, 95.2%), with one multinational study carried out in Italy and the USA. The proportion of male patients ranged from 53.1% to 94.7%, with a pooled mean of 72.8% (95% CI: 62.8–82.8). The reported mean patient age in the study cohorts ranged from 42.6 to 58.9 years.

**Table 1:** Characteristics of included studies.

**First Author et al. (year)**	**Country**	**Year**	**Study Design**	**Number of patients**	**Mean/Median age**	**Prevalence of male gender (%)**	**Treatment regimen**	**Tumor type**	**Tumor stage or grade**	**Magnetic field of strength (T)**	**MRI sequence**	**Response outcome classification**
Hu C et al. (2021)	China	2021	Retrospective Single-center	284	training:47.1 ± 11.3, test:47.0 ± 11.0	77.50%	Neoadjuvant chemotherapy	Nasopharyngeal carcinoma	III:161(56.7%),IVa:123(43.3%)	3 T	T1WI,T2WI	(CR/PR) vs. (SD/PD)
Huang L et al. (2023)	China	2023	Retrospective Single-center	65	44.85 ± 10.55 (responders), 44.00 ± 11.30 (non-responders)	72.30%	Recombinant human endostatin combined with concurrent chemoradiotherapy	Nasopharyngeal carcinoma	III:22(33.8%)–IVa:43(66.2%)	1.5 T	T2WI-FS,T1WI-CE	(CR/PR) vs. (SD/PD)
Yongfeng P et al. (2021)	China	2021	Retrospective Single-center	108	54 (22–70)	74.10%	neoadjuvant chemotherapy followed by chemoradiotherapy	Nasopharyngeal carcinoma	III: 48(44.4%)IV: 60(55.6%)	3 T	(T1W,T2W,T2W-FS)fast spin-echo,T1WI-CE	(CR/PR) vs. (SD/PD)
Wang Y et al. (2024)	China	2024	Retrospective Multi-center	389	center1: 51(IQR 46, 58), center2:51.5(IQR 45, 57), center 3:51.5 (IQR 46.5, 58)	72.30%	Neoadjuvant Chemotherapy	Nasopharyngeal carcinoma	II:23(5.9%),III:232(59.6%),IV:134(34.5%)	1.5 T&3 T	T1WI,T2WI,Thrive (T1-weighted high-resolution isotropic volume examination)	(CR/PR) vs. (SD/PD)
Pan M et al. (2025)	China	2025	Retrospective Single-center	265	42.60 ± 10.21	76.60%	Induction Chemotherapy	Mostly non-keratinizing squamous cell Nasopharyngeal carcinoma (92.39%)	III-Iva	1.5 T&3 T	T1WI-CE,T2-FS,DWI,DKI,IVIM,DCE-MRI	(CR) vs. (PR/SD/PD)
Bologna M et al. (2022)	Italy, USA	2022	Prospective Multi-center	50	54 ± 12 (34–78)	82%	induction chemotherapy (patients with resectable tumors undergo surgical resection if there is residual disease), Both groups (resectable and unresectable tumors) receive radiation therapy, with or without concurrent chemotherapy,	SNCs including SCC:12(24%),SNUC:23(46%),SNEC/ONB:11(22%) and ITAC:4(8%)	III:3(6%),IVa:20(40%),IVb:27(54%)	1.5 T&3 T	T1WI,T2WI,DWI and ADC maps	(CR/PR) vs. (SD/PD)
Yuan J et al. (2024)	China	2024	Retrospective Single-center	104	CR:55.09 ± 12.49, Non-CR: 52.92 ± 11.88	Training cohort: (CR:32 out of 44 (72.7%), Non-CR: 22 out of 26 (84.6%)), Test cohort: (CR:12 out of 19(63.2%), Non-CR: 10 out of 11 (90.9%))	Induction Chemotherapy followed by Concurrent Chemo Radiotherapy	Non-keratinizing Nasopharyngeal squamous cell carcinoma	III-Iva	3 T	T1WI,T1W-CE,T2WI,DWI,ADC	(CR) vs. (PR/SD/PD)
Wei H et al. (2025)	China	2025	Retrospective Single-center	49	CR:57 ± 5.0, non-CR:58 ± 10.8	CR:21/24:87.5%, non-CR:25/25:100%	Neoadjuvant chemoimmunotherapy	Locally Advanced Head and Neck Squamous Cell Carcinoma	Pathological grades:Poor:7 (29.2%) in CR, 11 (44.0%) in non-CR /Moderate: 14 (58.3%) in CR, 8 (32.0%) in non-CR/Well: 3 (12.5%) in CR, 6 (24.0%) in non-CR	3 T	T1WI, T1WI-CE, T2WI-FS,DWI	(CR) vs. (PR/SD/PD)
Wang G et al. (2018)	China	2018	Retrospective Single-center	120	46.81 ± 10.89 range (22–70)	79.20%	Induction chemotherapy	Nasopharyngeal carcinoma	II:10(8.3%),III:70(58.3%),IV:40(33.3%)	1.5 T	T1WI, T2WI, T1WI-CE, T2WI-FS	(CR/PR) vs. (SD/PD)
Guo Y et al. (2023)	China	2023	Retrospective Single-center	80	CR:46.60 ± 10.47, non-CR:52.82 ± 11.97	CR:80.7%, Non-CR:66.7%	Neoadjuvant or adjuvant chemotherapy and/or concurrent chemoradiotherapy with radiotherapy	Nasopharyngeal carcinoma	II:(CR:11.3%, non-CR:22.2%),III:(CR:62.9%, non-CR:22.2%),IV:(CR:25.8%, non-CR:55.6%)	3 T	DWI	(CR) vs. (PR/SD/PD)
Liu J et al. (2016)	China	2016	Prospective Single-center	53	Median:(Respoders:48(24–62), non-Responders:51(38–70))	Responders: 58.1%, non-Responders: 63.6%	Chemoradiotherapy	Nasopharyngeal carcinoma	not mentioned	3 T	T1WI, T2WI, DWI	(CR/PR) vs. (SD/PD)
Wang A et al. (2023)	China	2023	Retrospective Single-center	184	48.7(Primary cohort:48.9,Validation cohort:48.2)	70.5%(Primary cohort:69%, Validation cohort:74.2%)	Induction Chemotherapy	Nasopharyngeal carcinoma	II:30(16.3%),III:73(39.7%),IV:81(44%)	1.5 T&3 T	T2WI, T1WI-CE	(CR/PR) vs. (SD/PD)
Xu H et al. (2022)	China	2022	Retrospective Single-center	145	Response group [80]:46.06 ± 12.17, non-response group[65]:48.65 ± 13.07	80.7%:Response group [80]:80.0%, non-response group[65]:81.5%	Induction Chemotherapy followed by Concurrent Chemo Radiotherapy	Nasopharyngeal carcinoma	II:2(1.4%),III:59(40.7%),IV:84(57.9%)	1.5 T&3 T	T2WI, T1WI-CE	(CR/PR) vs. (SD/PD)
Liao H et al. (2022)	China	2022	Retrospective Single-center	286	43.7 ± 11.0	53.10%	Chemotherapy (TPF,GP regimen)	Locoregionally advanced nasopharyngeal carcinoma	III:86 (30.1%),IVa:200 (69.9%)	1.5 T	T1WI, T2WI, T1WI-CE	(CR/PR) vs. (SD/PD)
Liao H et al. (2025)	China	2025	Retrospective Multi-center	1368	46.7(H1(1.5 T):45,H1(3 T):46,H2:48,H3:47)	76.20%	Induction Chemotherapy Concurrent Chemoradiotherapy	Locoregionally advanced nasopharyngeal carcinoma	III,Iva	1.5 T&3 T	T2WI, T1WI-CE	(CR/PR) vs. (SD/PD)
Wang Y et al. (2025)	China	2025	Prospective Single-center	99	cCR:46(31–67), non-cCR:48(18–70)	76.80%	Induction Chemotherapy: PD-1 inhibitor and GP	Nasopharyngeal carcinoma associated with Epstein-Barr virus infection	III-IV	3 T	T2WI, T1WI-CE	(CR) vs. (PR/SD/PD)
Wang Y et al. (2022)	China	2022	Retrospective Single-center	165	respoders of (training:47.76 ± 12.13, testing:47.28 ± 11.42)	72.10%	Neoadjuvant Chemotherapy	Nasopharyngeal carcinoma	III,IVb	1.5 T	T1WI, T2WI, T1WI-CE	(CR/PR) vs. (SD/PD)
Qiu L et al. (2025)	China	2025	Retrospective Multi-center	104	not mentioned	not mentioned	Induction Chemotherapy followed by Concurrent Chemo Radiotherapy	Locally advanced nasopharyngeal carcinoma	III,Iva	3 T	T1WI, T2WI, T1WI-CE, DWI,ADC	(CR) vs. (PR/SD/PD)
Li Z et al. (2023)	China	2023	Retrospective Single-center	150	respoders of (training:58.88 ± 8.37 test:58.24 ± 7.23)	94.70%	Induction chemotherapy	Head and neck squamous cell carcinoma	II:11(7.3%),III:18(12%),IV:121(80.7%)	3 T	T2WI,pre- and post-CE-T1WI	(CR/PR) vs. (SD/PD)
Zhu Y et al. (2024)	China	2024	Retrospective Single-center	297	Median:45(13–90)	79.80%	Neoadjuvant chemotherapy	Nasopharyngeal carcinoma	II:6(2.0%),III:149(50.2%),IV:142(47.8%)	3 T	T1WI, T2WI-FS, FS-CE T1WI	(CR/PR) vs. (SD/PD)
Chen Z et al. (2024)	China	2024	Retrospective Multi-center	168	Median:52.5(IQR: 45.0–59.0)	59.50%	Induction Chemotherapy	Nasopharyngeal Carcinoma	II:5(3.0%),III:53 (31.5%), IV:110 (65.5%)	1.5 T	T1WI, T2WI-FS, CE-T1WI	(CR/PR) vs. (SD/PD)

Eighteen studies (85.7%) were retrospective, and three were prospective. Sixteen were conducted at a single center, and five were multicenter studies. Eighteen studies focused on nasopharyngeal carcinoma, including locally advanced, non-keratinizing, and EBV-associated subgroups; two assessed HNSCC, and the remaining one focused on sinonasal cancer.

Regarding tumor segmentation, all studies employed the manual method. A mix of magnetic field strengths was utilized, as ten studies used 3 T MRI exclusively, five used 1.5 T, and six included data from both. Imaging protocols primarily relied on conventional MRI sequences, with T1-weighted imaging (T1WI) used in 20 studies and T2-weighted imaging (T2WI) in 16. Diffusion-weighted imaging with ADC mapping was applied in ten studies. Advanced techniques such as dynamic contrast-enhanced MRI, intravoxel incoherent motion, and diffusion kurtosis imaging were reported in only four studies.

The most prevalent treatment strategy was induction or neoadjuvant chemotherapy (IC/NACT), reported in 15 studies. This was administered either as a standalone therapy (n = 9) or in combination with subsequent concurrent chemoradiotherapy (CCRT) (n = 6). Other therapeutic approaches included chemoimmunotherapy (n = 2), chemotherapy alone (n = 1), CCRT alone (n = 1), and other mixed or novel regimens (n = 2). Detailed data regarding the radiomics pipeline in the included studies can be found in the Supplementary material.

### 3.3 Methodological quality assessment

The quality of the included studies was high in general according to the METRICS scale. The mean METRICS score across the 21 studies was 80.67% ± 7.00% (range, 66.50–92.60%). According to established METRICS criteria [27], the majority of the studies (14/21, 66.7%) were rated as 'excellent' (score ≥ 80%), and the remaining seven (33.3%) were classified as 'good' (60 ≤ score < 80%).

Despite high METRICS scores, several key methodological weaknesses were identified. A primary limitation was the lack of model generalizability, as external validation was performed in only three studies [30], [37], [41]. Furthermore, model calibration was often inadequately addressed. The potential risk of model overfitting was noted, as several studies utilized sample sizes relatively small in comparison to the number of selected radiomic features. Also, the comparative value of specific MRI sequences has been neglected due to the general lack of uni-parametric analysis, and the radiomics methodology has been predominantly formed by traditional ML, with minimal use of deep learning models. Detailed parameters for the metrics assessment of each study can be found in the Supplementary material.

### 3.4 Meta-analysis of predictive performance

We conducted meta-analyses to evaluate the diagnostic accuracy of radiomics models for predicting PR/CR in comparison to PD/SD, and also CR against other types of response (PR/SD/PD). A comprehensive summary of the diagnostic performance metrics is provided in Table 2**.**

**Table 2:** Summarized predictive performance of radiomics and radiomics + clinical models in differentiating treatment response of HNSCC patients. AUC: area under the curve, PLR: positive likelihood ratio, NLR: negative likelihood ratio, DOR: diagnostic odds ratio, CR: complete response, PR: partial response, SD: stable disease, PD: progressive disease.

	No. of studies	Pooled Sensitivity	Pooled Specificity	AUC	PLR	NLR	DOR	Heterogeneity (I²)
		(95% CI)	(95% CI)	(95% CI)	(95% CI)	(95% CI)	(95% CI)	
CR/PR vs PD/SD								
Radiomics								
Training	11	82% (76%,87%)	84% (73%,90%)	0.89 (0.86,0.92)	5.0 (3.1,8.1)	0.21 (0.16,0.28)	24 (14,40)	74.5%
Validation	14	79% (72%,84%)	76% (68%,83%)	0.84 (0.81,0.87)	3.3 (2.5,4.4)	0.28 (0.21,0.37)	12 (8,19)	56.5%
Radiomics + Clinical								
Training	7	84% (76%,89%)	78% (73%,83%)	0.86 (0.83,0.89)	3.9 (2.9,5.2)	0.21 (0.13,0.32)	19 (9,37)	51.5%
Validation	8	79% (73%,85%)	72% (62%,80%)	0.83 (0.80,0.86)	2.9 (2.1,3.8)	0.28 (0.22,0.37)	10 (7,15)	0.00%
CR vs PR/PD/SD								
Radiomics								
Training	5	89% (80%,94%)	86% (76%,93%)	0.94 (0.91,0.96)	6.5 (3.7,11.7)	0.13 (0.07,0.23)	50 (26,97)	0.06%
Validation	5	68% (55%,79%)	87% (74%,94%)	0.83 (0.79,0.86)	5.2 (2.7,10.0)	0.37 (0.26,0.51)	14 (7,29)	0.02%

#### 3.4.1 Prediction of partial or complete response (PR/CR)

**Fig. 2:**
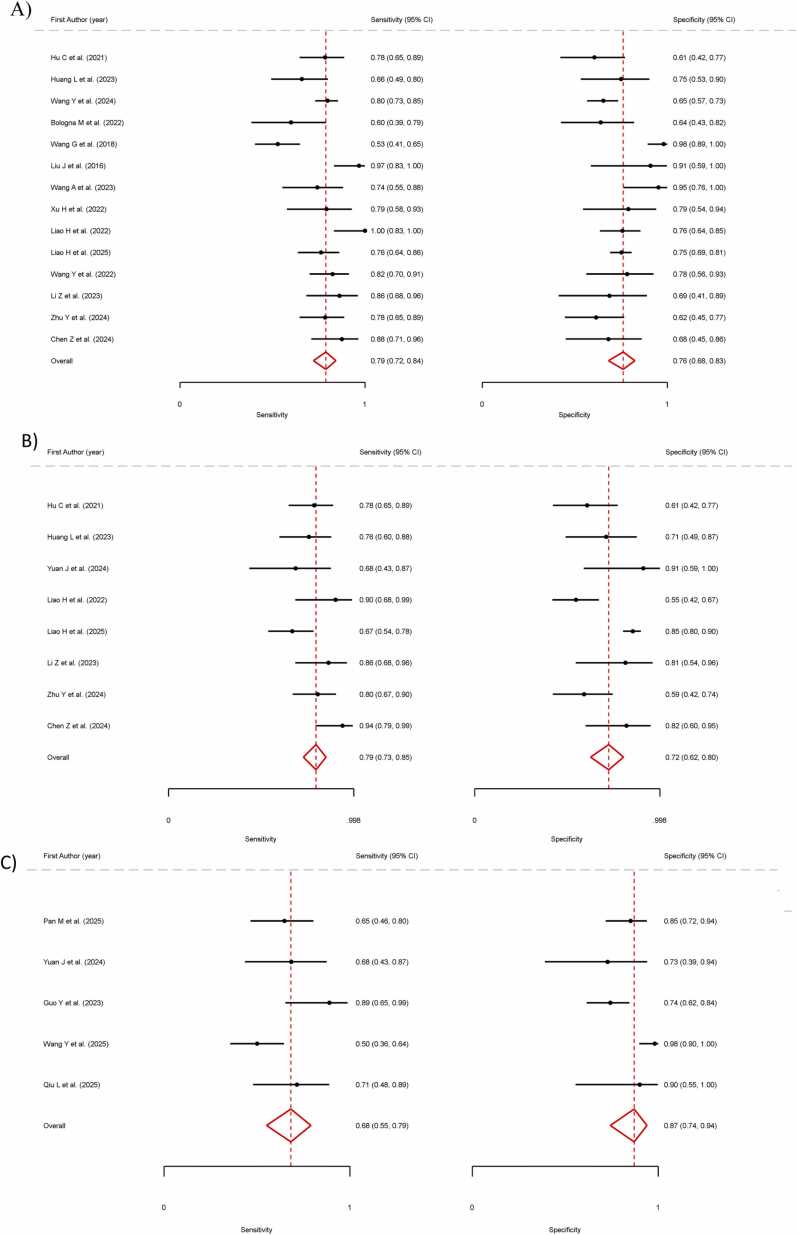
Meta-analysis of predictive performance for the validation cohorts. Forest plots display the pooled sensitivity and specificity for two classification tasks: (A) distinguishing responders (CR/PR) from non-responders (PD/SD) in radiomics-only model, (B) distinguishing responders (CR/PR) from non-responders (PD/SD) in combine model and, (C) distinguishing a Complete Response (CR) from all other outcomes (PR/SD/PD) in radiomics-only model. Individual study estimates are represented by black dots with corresponding 95% confidence intervals. The overall pooled estimates and their confidence intervals are indicated by the red diamonds.

For radiomics-only models, the meta-analysis of 11 training cohorts yielded a pooled sensitivity of 0.82 (95% CI: 0.76–0.87) and a specificity of 0.84 (95% CI: 0.73–0.90), with a pooled AUC of 0.89 (95% CI: 0.86–0.92). However, significant heterogeneity was observed (I² = 74.5%). For the corresponding 14 validation studies, the pooled sensitivity was 0.79 (95% CI: 0.72–0.84) and specificity was 0.76 (95% CI: 0.68–0.83), with a pooled AUC of 0.84 (95% CI: 0.81–0.87), with moderate heterogeneity (I² = 56.5%) **(**Fig. 2**A)**.

**Fig. 3:**
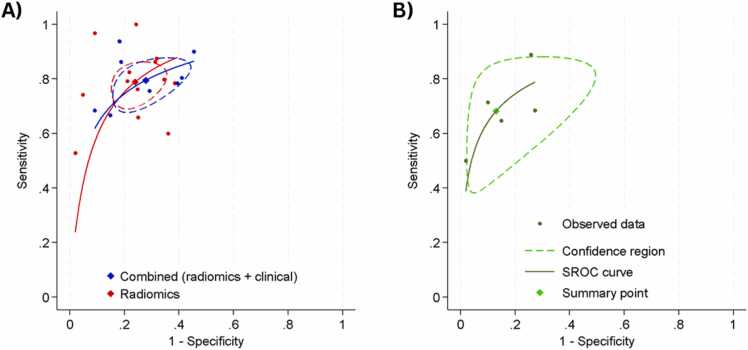
Summary receiver operating characteristic (SROC) curves for the validation of treatment response models. (A) Compares a radiomics-only model (red) with a combined radiomics-clinical model (blue) for distinguishing responders (CR/PR) from non-responders (PD/SD), and (B) shows the performance of the radiomics-only model for distinguishing a Complete Response (CR) from all other response categories (PR/SD/PD).

For combined models (radiomics + clinical data) a pooled sensitivity of 0.84 (95% CI: 0.76–0.89) and a specificity of 0.78 (95% CI: 0.73–0.83), with a pooled AUC of 0.86 (95% CI: 0.83–0.89) was determined in meta-analysis of seven training sets. Also, eight eligible studies reporting on validation cohorts were analyzed. The pooled sensitivity was 0.79 (95% CI: 0.73–0.85) and specificity was 0.72 (95% CI: 0.62–0.80) **(**Fig. 2**B)**. The pooled AUC was 0.83 (95% CI: 0.80–0.86), with no evidence of heterogeneity (I² = 0.00%). A meta-analysis on external validation cohorts could not be performed, as only three studies provided the necessary data. The SROC curves, visually summarizing the comparative performance of the combined and radiomics-only validation models, are presented in Fig. 3**A**.

To investigate sources of the observed heterogeneity (I² = 56.5%) among validation cohorts of radiomics-only models, subgroup analyses were conducted (Table 3). High levels of heterogeneity were confined to single-center studies (I² = 62.00%) and those evaluating NACT followed by CCRT (I² = 69.05%). In contrast, multi-center studies, studies assessing NACT alone, and models developed using 3 T MRI scans exclusively or a combination of 1.5 T and 3 T scanners, all demonstrated complete homogeneity (I² = 0.0%), indicating highly consistent results. A meta-regression analysis was conducted to assess the impact of study-level covariates on model performance. Neither the patient gender (p = 0.142) nor the METRICS score (p = 1.000) showed a significant effect. Furthermore, a leave-one-out sensitivity analysis confirmed that no single study disproportionately influenced the pooled estimates in radiomics-only validation set results. Deeks’ funnel plot asymmetry test showed no evidence of publication bias for predicting PR/CR (p = 0.20) **(**Supplementary material**).**

**Table 3:** Subgroup analyses of performance for the radiomics-only validation models in distinguishing responders (CR/PR) from non-responders (PD/SD).

Subgroup	No. of Studies	Pooled Sensitivity	Pooled Specificity	Heterogeneity (I²)
		(95% CI)	(95% CI)	
Study Design				
Retrospective	12	0.77 (0.72,0.82)	0.76 (0.67,0.83)	00.0%
Prospective	2	0.80 (0.68,0.89)	0.72 (0.56,0.84)	N/A
Study Setting				
Single center	10	0.80 (0.71,0.87)	0.80 (0.69,0.88)	62.00%
Multi-center	4	0.78 (0.72,0.83)	0.70 (0.64,0.76)	00.0%
Treatment Regimen				
NACT	8	0.82 (0.77,0.85)	0.70 (0.64,0.76)	00.0%
NACT + CCRT	6	0.74 (0.59,0.85)	0.83 (0.68,0.91)	69.05%
MRI Field Strength				
1.5 T	5	0.79 (0.62,0.90)	0.83 (0.69,0.91)	52.0%
3 T	4	0.86 (0.74,0.93)	0.69 (0.53,0.82)	00.0%
1.5 T & 3 T	5	0.76 (0.70,0.81)	0.74 (0.65,0.81)	00.0%

#### 3.4.2 Prediction of complete response (CR)

To evaluate the accuracy of radiomics-only models in predicting CR vs other outcomes (PR/SD/PD), a meta-analysis of five eligible studies was conducted. For the training cohorts, the pooled sensitivity was 0.89 (95% CI: 0.80–0.94) and the pooled specificity was 0.86 (95% CI: 0.76–0.93). For the corresponding validation cohorts, the pooled sensitivity was 0.68 (95% CI: 0.55–0.79) and specificity was 0.87 (95% CI: 0.74–0.94) with negligible heterogeneity (I² = 0.02%), as detailed in the forest plot in Fig. 2**C**. The diagnostic performance is summarized by the SROC curve in Fig. 3**B**. Sensitivity analysis showed no outlier study, and Deeks' funnel plot test revealed no significant publication bias (p = 0.36) **(**Supplementary material**).** A meta-analysis of combined radiomics-clinical models could not be performed, as only two studies provided eligible data for this endpoint [33], [44].

### 3.5 Certainty of evidence

The results of this study, summarized in Table 4, were assessed using the GRADE tool to inform policy-making and integration into and clinical contexts. GRADE assessment determined a low to moderate certainty of the evidence for all subsections of results. This rating was based on a sufficient number of included studies, a moderate to high risk of bias and indirectness (mainly due to retrospective single-center design and lack of external validation), low heterogeneity (or resolved heterogeneity where initially present), an absence of significant publication bias, and the acceptable AUC values of the models.

**Table 4:** Summary findings profile based on the grading of recommendations, assessment, development, and evaluations (GRADE).

Outcome	Number of studies	Diagnostic results	Risk of bias	Applicability and indirectness	Inconsistency	Imprecision	Publication bias	Strength of effect size	Certainty
CR/PR vs PD/SD Radiomics model in validation set	14	Fig. 2, Fig. 3Supplementary material	Moderate risk	High risk	Inconsistent	Precise	Low risk	High Strength	Low ⊕⊕
CR/PR vs PD/SD Combine model in validation set	8	Fig. 2, Fig. 3Supplementary material	Moderate risk	High risk	Consistent	Precise	Low risk	High Strength	Moderate ⊕⊕⊕
CR vs PR/PD/SD Radiomics model in validation set	5	Fig. 2, Fig. 3Supplementary material	Moderate risk	High risk	Consistent	Precise	Low risk	High Strength	Moderate ⊕⊕⊕

## 4 Discussion

This work represents a comprehensive meta-analysis of MRI-based radiomics studies for the prediction of treatment response in HNSCC patient. Intratumoral heterogeneity is a significant hurdle in HNSCC treatment, with up to 23.1% of nasopharyngeal carcinomas resistant to chemotherapy [48]. Our pooled results demonstrate that MRI radiomics is a powerful tool for response prediction, with pooled AUCs of 0.83 for differentiating complete responders from other outcomes and 0.84 for distinguishing (CR/PR) from (SD/PD). Notably, the high performance of radiomics was consistent across these various response outcome definitions with no publication bias and no outlier study, which emphasizes robustness of MRI radiomics as a biomarker for response assessment. Identifying non-responders could guide treatment intensification through combined immunotherapy, targeted agents, optimized maintenance regimens, or closer surveillance [41], [44]. However, while MRI radiomics holds promise for advancing precision oncology in HNSCC, several critical methodological and validation issues must be addressed before its routine incorporation into clinical practice can be realized.

Subgroup analysis of radiomics models for differentiating (CR/PR) from (SD/PD), as the most frequently reported outcome in our meta-analysis, demonstrated consistent predictive performance across all clinical scenarios examined with no meaningful difference between subgroups. Also, subgroup analyses based on treatment regimen explained a portion of the observed heterogeneity. Heterogeneity remained high in the subgroup of patients receiving NACT followed by CCRT (I^2^ = 69.05%), while the NACT-only group showed no significant heterogeneity (I^2^ = 0.00%). This discrepancy could be due to the greater variability in CCRT protocols and the more complex, heterogeneous biological responses induced by combined modality therapy. Additionally, variations in chemotherapy agents, treatment cycles, radiation doses, fractionation schedules, overall treatment durations, and sequencing across different treatment centers likely contributed to the observed heterogeneity. Furthermore, additional subgroup analyses confirmed that methodological factors, specifically study design and magnetic field strength, also accounted for a portion of the heterogeneity. The pooled estimates for validation set performance in our results indicate strong reliability as shown by consistently low heterogeneity, with any remaining variability addressed through subgroup analysis. The results of the subgroup analyses should be interpreted with caution and considered primarily hypothesis‑generating. Several subgroups contained a limited number of studies, which reduces the statistical power and stability of pooled estimates. In such circumstances, both the observed differences between subgroups and the calculated heterogeneity may be imprecise and potentially influenced by random variation rather than reflecting true underlying effects.

Our findings on performance of radiomics and combined models, frequently showed higher performance of training cohorts in comparison to the validation cohorts. This issue is a well-established indicator of model overfitting, a major risk in radiomics research where several features are frequently extracted from a restricted sample size. In these situations, models may acquire the noise and particular biases of the training dataset instead of general biological patterns, resulting into inflated optimistic performance metrics [49]. Thus, the performance metrics obtained from the validation cohorts offer a more realistic and clinically relevant assessment of model efficacy compared to those from the training sets. It is noteworthy that the three studies conducting external validation indicated no substantial drop in model performance with their upper confidence limits fell within the 95% CI of our pooled validation AUC [30], [37], [41]. They reported AUCs ranging from 0.672 to 0.832 for radiomics-only and combined models, with relatively wide confidence intervals reflecting their small sample sizes. This initial finding possibly supports the generalizability of the radiomics technique, however it requires substantially more validation through future large, multi-center studies.

Interestingly, the addition of clinical variables to radiomics models did not significantly improve predictive performance. The AUC was 0.84 for radiomics-only models, compared to 0.83 for combined models. This finding diverges from a recent meta-analysis on the role of radiomics in predicting overall survival in this patient group [50]. Several factors may explain this observation. First, the clinical variables used across the analyzed studies included a wide range of parameters such as age, sex, T stage, LDH level, induction chemotherapy regimens, neutrophil count, and EBV-DNA [28], [40], [41], [46], [47]. This lack of uniformity complicates direct comparisons and can dilute the collective predictive power of clinical data. Second, radiomics signature may capture, to a substantial extent, the biological and prognostic information represented by the included clinical factors. Consequently, the added value of these clinical variables may not be a statistically significant [47]. Also, radiomics can quantify underlying tumor characteristics such as proliferation, cellularity, hypoxia, and necrosis, which are often linked to poor outcomes [51]. For instance, in nasopharyngeal carcinoma, conventional clinical staging may group biologically different tumors with distinct prognoses [52]. Finally, in some of the included studies, clinical variables showed moderate to poor discrimination, lost significance in multivariable analyses when radiomic features were included, or showed moderate to low predictive efficiency compared to radiomics models [29], [40], [47]. Some clinical predictors demonstrated prognostic value only in short-term or in subset outcomes, which may explain the weaker clinical-only model performance [45]. However, although the pooled AUCs were similar, the heterogeneity of the combined models was substantially lower (0% vs 56.5%), suggesting better generalizability and robustness. This may be partly explained by the relatively greater similarity of the clinical variables used across studies, whereas radiomics-only models relied on highly heterogeneous feature sets, leading to greater variability between studies. Importantly, this lower heterogeneity together with an acceptable AUC supports the potential value of combined models as more stable and generalizable predictive tools.

Many included studies underscored the superiority of multisequence radiomics models over those based on a single MRI sequence [30], [32], [35], [44]. Despite the common use of conventional T1-weighted (T1W) and T2-weighted (T2W) imaging, one study by Qiu et al. demonstrated their inferiority to contrast-enhanced T1-weighted (CE-T1W), ADC, and DWI sequences [44]. While the ADC and DWI parameter was widely used in many studies, four studies specifically demonstrated the added value of ADC sequences in models exclusively based on ADC, reporting their performance compared with models derived from other MRI sequences [32], [33], [37], [44]. Although multi-sequence model development results in higher model performance, uni-parametric analyses are particularly important for determining the relative contribution of individual MRI sequences in ML model development; however, nearly all included studies constructed models using the entire set of available MRI sequences, often based on heterogeneous acquisition protocols and sequence combinations, without evaluating the independent impact of each sequence. Consequently, the current evidence does not allow reliable subgroup analyses and conclusions regarding the comparative value of conventional vs functional MRI sequences. In addition to MRI-based models, the performance of PET/CT-based radiomics models highlights the value of multimodal data. For instance, one study achieved a sensitivity of 93% and a specificity of 83%, suggesting that metabolic information from PET provides complementary predictive value beyond anatomical features alone [53]. These findings emphasize the importance of integrating both functional and metabolic imaging data to enhance predictive model performance. Therefore, future research should prioritize the inclusion of advanced functional and multimodal imaging sequences to build more robust and clinically applicable models. Beyond response prediction, radiomics also offers valuable prognostic information. This capability is supported by several meta-analyses, demonstrating that MRI- and PET/CT-based models can predict overall survival with pooled C-indices ranging from 0.68 to 0.77, particularly when clinical variables are incorporated [50], [54].

A critical observation is that more sophisticated modeling approaches, such as graph neural networks (GNNs) and backpropagation neural networks (BPNNs), yielded superior performance compared to conventional ML models [40], [41], suggesting that the conventional ML models may be inadequate for fully capturing the complex, non-linear relationship between radiomic features, clinical variables, and treatment outcomes [41]. However, the limited number of studies directly comparing deep learning models to handcrafted radiomics (HCR) techniques prevented a formal meta-analysis on this direct comparison topic [42]. Future research should prioritize robust, multi-center studies with direct head-to-head comparisons conventional ML, and advanced DL models, including convolutional neural networks (CNNs) for end-to-end feature extraction, to establish the optimal analytical framework.

Although the meta-analysis revealed promising results, several methodological concerns limit the robustness and generalizability of the conclusions. While we intended to include all HNSCC subsites, the available evidence was heavily skewed toward nasopharyngeal cancer, with 18 of the 21 included studies focusing on this subsite, creating a mismatch between the broader HNSCC scope and the underlying evidence base. Furthermore, the evidence base exhibits a significant geographic concentration, as nearly all included studies were conducted within China. This regional homogeneity inherently restricts the external validation and generalizability of the findings across the full global spectrum of HNSCC patients and healthcare systems, who may present with distinct genetic, environmental, and clinical profiles. In addition, our METRICS evaluation showed that, important limitations remained: external validation was performed in only a few studies, model calibration was often insufficiently reported, and the relatively small sample sizes compared with the number of extracted radiomic features raise concerns regarding potential overfitting. Consistent with mentioned issues, the GRADE assessment indicated overall low-to-moderate certainty of evidence, precluding robust conclusions despite the encouraging results. Future research must focus on the development of standardized radiomics pipelines and validate models across multi-center cohorts to achieve a high certainty level in the evidence. Methodological limitations were further reflected by the limited use of uni-parametric analyses to assess the comparative value of specific MRI sequences and the predominant reliance on traditional ML approaches, with minimal exploration of deep learning models.

Moreover, considerable variability in imaging protocols and field strength and inconsistent preprocessing or feature-selection workflows may have introduced methodological heterogeneity in model training. To address this, future research must focus on the development of standardized radiomics pipelines and validate models across multi-center cohorts [46]. All included studies consistently used RECIST criteria as the reference standard, which assess radiologic dimensional changes rather than true pathological or molecular tumor viability. This may lead to underestimation of pathological complete response and potential verification bias, especially in non-surgical cohorts. However, we adopted the RECIST framework given its widespread use and dominance in the existing literature.

In conclusion, this systematic review and meta-analysis indicate that MRI-based radiomics serves as a robust tool for assessing treatment response to NACT in HNSCC. The radiomics model demonstrated consistently high predictive performance for differentiating responders from non-responders across two relevant response definition. This performance remained stable in subgroup analyses, by variations in therapeutic regimens, MRI magnetic field strength, or study methodology, underscoring its reliability. Furthermore, combined models that integrate radiomic features with clinical data showed good performance. However, given the predominantly retrospective nature of the available studies, their geographic concentration, and the overall low-to-moderate quality of evidence, our findings should be interpreted with caution. While MRI-based radiomics shows potential as a non-invasive biomarker for treatment stratification in HNSCC, further methodological standardization and large, prospective, multicenter studies with robust external validation are required before meaningful clinical integration can be considered.

## CRediT authorship contribution statement

**Saeed Mohammadzadeh:** Writing – review & editing, Writing – original draft, Project administration, Methodology, Investigation, Formal analysis, Data curation, Conceptualization. **Sajad Mohammadzadeh:** Writing – review & editing, Methodology, Data curation. **Seyed Amir Mohammad Seyed Rahmani:** Writing – original draft, Data curation. **Iman Kiani:** Writing – review & editing, Methodology, Formal analysis. **Fatemeh Mahdavi Sabet:** Writing – review & editing, Writing – original draft. **Houman Sotoudeh:** Validation, Supervision, Project administration. **Farzad Fayedeh:** Writing – review & editing, Data curation.

## Consent to participate

All participants provided written informed consent.

## Animal study

N/A.

## Funding

None.

## Declaration of Competing Interest

The authors declare that they have no known competing financial interests or personal relationships that could have appeared to influence the work reported in this paper.

## References

[1] Mody M.D., Rocco J.W., Yom S.S., Haddad R.I., Saba N.F. (2021). Head and neck cancer. Lancet.

[2] Bolnykh I., Patterson J.M., Harding S., Watson L.-J., Lu L., Hurley K. (2025). Cancer-related pain in head and neck cancer survivors: longitudinal findings from the Head and Neck 5000 clinical cohort. J. Cancer Surviv..

[3] Schoenberg P., Wulff-Burchfield E., Schlundt D., Bonnet K., Dietrich M., Murphy B. (2024). Qualitative classification of late systemic symptoms in head and neck cancer survivors. Cancers.

[4] Forastiere A.A., Trotti A., Pfister D.G., Grandis J.R. (2006). Head and neck cancer: recent advances and new standards of care. J. Clin. Oncol..

[5] Barsouk A., Aluru J.S., Rawla P., Saginala K., Barsouk A. (2023). Epidemiology, risk factors, and prevention of head and neck squamous cell carcinoma. Med. Sci..

[6] Peng Z., Wang Y., Wang Y., Jiang S., Fan R., Zhang H. (2021). Application of radiomics and machine learning in head and neck cancers. Int. J. Biol. Sci..

[7] Zorat P.L., Paccagnella A., Cavaniglia G., Loreggian L., Gava A., Mione C.A. (2004). Randomized phase III trial of neoadjuvant chemotherapy in head and neck cancer: 10-year follow-up. J. Natl. Cancer Inst..

[8] Paccagnella A., Orlando A., Marchiori C., Zorat P.L., Cavaniglia G., Sileni V.C. (1994). Phase III trial of initial chemotherapy in stage III or IV head and neck cancers: a study by the Gruppo di Studio sui Tumori della Testa e del Collo. JNCI: J. Natl. Cancer Inst..

[9] Zhang X.-r., Liu Z.-m., Liu X.-k., Wang F.-h., Li Q., Li H. (2013). Influence of pathologic complete response to neoadjuvant chemotherapy on long-term survival of patients with advanced head and neck squamous cell carcinoma. Oral Surg. Oral Med. Oral Pathol. Oral Radiol..

[10] Posner M.R. (2005). Paradigm shift in the treatment of head and neck cancer: the role of neoadjuvant chemotherapy. Oncologist.

[11] Giraud P., Giraud P., Gasnier A., El Ayachy R., Kreps S., Foy J.P. (2019). Radiomics and machine learning for radiotherapy in head and neck cancers. Front. Oncol..

[12] Lambin P., Leijenaar R.T., Deist T.M., Peerlings J., De Jong E.E., Van Timmeren J. (2017). Radiomics: the bridge between medical imaging and personalized medicine. Nat. Rev. Clin. Oncol..

[13] Lambin P., Rios-Velazquez E., Leijenaar R., Carvalho S., Van Stiphout R.G., Granton P. (2012). Radiomics: extracting more information from medical images using advanced feature analysis. Eur. J. Cancer.

[14] Ng C.K., Pemberton H.N., Reis-Filho J.S. (2012). Breast cancer intratumor genetic heterogeneity: causes and implications. Expert. Rev. Anticancer. Ther..

[15] Szerlip N.J., Pedraza A., Chakravarty D., Azim M., McGuire J., Fang Y. (2012). Intratumoral heterogeneity of receptor tyrosine kinases EGFR and PDGFRA amplification in glioblastoma defines subpopulations with distinct growth factor response. Proc. Natl. Acad. Sci..

[16] Parmar C., Grossmann P., Rietveld D., Rietbergen M.M., Lambin P., Aerts H.J. (2015). Radiomic machine-learning classifiers for prognostic biomarkers of head and neck cancer. Front. Oncol..

[17] Peng Z., Wang Y., Wang Y., Jiang S., Fan R., Zhang H. (2021). Application of radiomics and machine learning in head and neck cancers. Int. J. Biol. Sci..

[18] Gonçalves M., Gsaxner C., Ferreira A., Li J., Puladi B., Kleesiek J. (2022). Radiomics in head and neck cancer outcome predictions. Diagnostics.

[19] Giannitto C., Mercante G., Ammirabile A., Cerri L., De Giorgi T., Lofino L. (2023). Radiomics-based machine learning for the diagnosis of lymph node metastases in patients with head and neck cancer: systematic review. Head Neck.

[20] Gul M., Bonjoc K.-J.C., Gorlin D., Wong C.W., Salem A., La V. (2021). Diagnostic utility of radiomics in thyroid and head and neck cancers. Front. Oncol..

[21] Jethanandani A., Lin T.A., Volpe S., Elhalawani H., Mohamed A.S.R., Yang P. (2018). Exploring applications of radiomics in magnetic resonance imaging of head and neck cancer: a systematic review. Front. Oncol..

[22] Hu C., Zheng D., Cao X., Pang P., Fang Y., Lu T. (2021). Application value of magnetic resonance radiomics and clinical nomograms in evaluating the sensitivity of neoadjuvant chemotherapy for nasopharyngeal carcinoma. Front. Oncol..

[23] Huang L., Yang Z., Kang M., Ren H., Jiang M., Tang C. (2023). Performance of pretreatment MRI-based radiomics in recombinant human endostatin plus concurrent chemoradiotherapy response prediction in nasopharyngeal carcinoma: a retrospective study. Technol. Cancer Res. Treat..

[24] Pan M., Lu L., Mu X., Jin G. (2025). Prediction of induction chemotherapy efficacy in patients with locally advanced nasopharyngeal carcinoma using habitat subregions derived from multi-modal MRI radiomics. Front. Oncol..

[25] McInnes M.D., Moher D., Thombs B.D., McGrath T.A., Bossuyt P.M., Clifford T. (2018). Preferred reporting items for a systematic review and meta-analysis of diagnostic test accuracy studies: the PRISMA-DTA statement. Jama.

[26] Rethlefsen M.L., Kirtley S., Waffenschmidt S., Ayala A.P., Moher D., Page M.J. (2021). PRISMA-S: an extension to the PRISMA statement for reporting literature searches in systematic reviews. Syst. Rev..

[27] Kocak B., Akinci D′Antonoli T., Mercaldo N., Alberich-Bayarri A., Baessler B., Ambrosini I. (2024). METhodological RadiomICs Score (METRICS): a quality scoring tool for radiomics research endorsed by EuSoMII. Insights Imaging.

[28] Hu C., Zheng D., Cao X., Pang P., Fang Y., Lu T. (2021). Application value of magnetic resonance radiomics and clinical nomograms in evaluating the sensitivity of neoadjuvant chemotherapy for nasopharyngeal carcinoma. Front. Oncol..

[29] Yongfeng P., Chuner J., Lei W., Fengqin Y., Zhimin Y., Zhenfu F. (2021). The usefulness of pretreatment MR-based radiomics on early response of neoadjuvant chemotherapy in patients with locally advanced nasopharyngeal carcinoma. Oncol. Res..

[30] Wang Y., Zhang H., Wang H., Hu Y., Wen Z., Deng H. (2024). Development of a neoadjuvant chemotherapy efficacy prediction model for nasopharyngeal carcinoma integrating magnetic resonance radiomics and pathomics: a multi-center retrospective study. BMC Cancer.

[31] Pan M., Lu L., Mu X., Jin G. (2025). Prediction of induction chemotherapy efficacy in patients with locally advanced nasopharyngeal carcinoma using habitat subregions derived from multi-modal MRI radiomics. Front. Oncol..

[32] Bologna M., Calareso G., Resteghini C., Sdao S., Montin E., Corino V. (2022). Relevance of apparent diffusion coefficient features for a radiomics-based prediction of response to induction chemotherapy in sinonasal cancer. NMR Biomed..

[33] Yuan J., Wu M., Qiu L., Xu W., Fei Y., Zhu Y. (2024). Tumor habitat-based MRI features assessing early response in locally advanced nasopharyngeal carcinoma. Oral Oncol..

[34] Wei H., Wang K., Yang F., Li X., Yu X., Zhao Y. (2025). MRI-based texture analysis for the evaluation of the response to neoadjuvant chemoimmunotherapy in locally advanced head and neck squamous cell carcinoma. BMC Med. Imaging.

[35] Wang G., He L., Yuan C., Huang Y., Liu Z., Liang C. (2018). Pretreatment MR imaging radiomics signatures for response prediction to induction chemotherapy in patients with nasopharyngeal carcinoma. Eur. J. Radiol..

[36] Guo Y., Dai G., Xiong X., Wang X., Chen H., Zhou X. (2023). Intravoxel incoherent motion radiomics nomogram for predicting tumor treatment responses in nasopharyngeal carcinoma. Transl. Oncol..

[37] Liu J., Mao Y., Li Z., Zhang D., Zhang Z., Hao S. (2016). Use of texture analysis based on contrast-enhanced MRI to predict treatment response to chemoradiotherapy in nasopharyngeal carcinoma. J. Magn. Reson. Imaging.

[38] Wang A., Xu H., Zhang C., Ren J., Liu J., Zhou P. (2023). Radiomic analysis of MRI for prediction of response to induction chemotherapy in nasopharyngeal carcinoma patients. Clin. Radiol..

[39] Xu H., Liu J., Huang Y., Zhou P., Ren J. (2021). MRI-based radiomics as response predictor to radiochemotherapy for metastatic cervical lymph node in nasopharyngeal carcinoma. Br. J. Radiol..

[40] Liao H., Chen X., Lu S., Jin G., Pei W., Li Y. (2022). MRI-based back propagation neural network model as a powerful tool for predicting the response to induction chemotherapy in locoregionally advanced nasopharyngeal carcinoma. J. Magn. Reson. Imaging.

[41] Liao H., Zhao Y., Pei W., Huang X., Huang S., Wei W. (2025). An interpretable machine learning model assists in predicting induction chemotherapy response and survival for locoregionally advanced nasopharyngeal carcinoma using MRI: a multicenter study. Eur. Radiol..

[42] Wang Y., Chen F., Ouyang Z., He S., Qin X., Liang X. (2025). MRI-based deep learning and radiomics for predicting the efficacy of PD-1 inhibitor combined with induction chemotherapy in advanced nasopharyngeal carcinoma: a prospective cohort study. Transl. Oncol..

[43] Wang Y., Li C., Yin G., Wang J., Li J., Wang P. (2022). Extraction parameter optimized radiomics for neoadjuvant chemotherapy response prognosis in advanced nasopharyngeal carcinoma. Clin. Transl. Radiat. Oncol..

[44] Qiu L., Fei Y., Zhu Y., Yuan J., Shi K., Wu M. (2025). Radiomic analysis based on machine learning of multi-sequences MR to assess early treatment response in locally advanced nasopharyngeal carcinoma. Sci. Prog..

[45] Li Z., Wang R., Wang L., Tan C., Xu J., Fang J. (2024). Machine learning-based MRI radiogenomics for evaluation of response to induction chemotherapy in head and neck squamous cell carcinoma. Acad. Radiol..

[46] Zhu Y., Zheng D., Xu S., Chen J., Wen L., Zhang Z. (2024). Intratumoral habitat radiomics based on magnetic resonance imaging for preoperative prediction treatment response to neoadjuvant chemotherapy in nasopharyngeal carcinoma. Jpn. J. Radiol..

[47] Chen Z., Wang Z., Liu S., Zhang S., Zhou Y., Zhang R. (2024). Nomograms based on multiparametric MRI radiomics integrated with clinical-radiological features for predicting the response to induction chemotherapy in nasopharyngeal carcinoma. Eur. J. Radiol..

[48] Liu S.L., Sun X.S., Yan J.J., Chen Q.Y., Lin H.X., Wen Y.F. (2019). Optimal cumulative cisplatin dose in nasopharyngeal carcinoma patients based on induction chemotherapy response. Radiother. Oncol..

[49] Moskowitz C.S., Welch M.L., Jacobs M.A., Kurland B.F., Simpson A.L. (2022). Radiomic analysis: study design, statistical analysis, and other bias mitigation strategies. Radiology.

[50] Wang T.W., Wang C.K., Hong J.S., Lin Y.H., Wang S.Y., Lu C.F. (2025). Prognostic power of radiomics in head and neck cancers: insights from a meta-analysis. Comput. Methods Prog. Biomed..

[51] Ganeshan B., Goh V., Mandeville H.C., Ng Q.S., Hoskin P.J., Miles K.A. (2013). Non-small cell lung cancer: histopathologic correlates for texture parameters at CT. Radiology.

[52] Chen F.P., Lin L., Qi Z.Y., Zhou G.Q., Guo R., Hu J. (2017). Pretreatment nomograms for local and regional recurrence after radical radiation therapy for primary nasopharyngeal carcinoma. J. Cancer.

[53] Zhang Q., Wang K., Zhou Z., Qin G., Wang L., Li P. (2022). Predicting local persistence/recurrence after radiation therapy for head and neck cancer from PET/CT using a multi-objective, multi-classifier radiomics model. Front. Oncol..

[54] Wang B., Liu J., Xie J., Zhang X., Wang Z., Cao Z. (2024). Systematic review and meta-analysis of the prognostic value of (18)F-Fluorodeoxyglucose ((18)F-FDG) positron emission tomography (PET) and/or computed tomography (CT)-based radiomics in head and neck cancer. Clin. Radiol.

