# Supplementary material for: Performance of magnetic-resonance imaging radiomics in prediction of response after neoadjuvant chemotherapy in head and neck squamous cell carcinoma: A systematic review and meta-analysis
Source: Eur J Radiol Open. 2026 Jun 22;17:100786. doi: 10.1016/j.ejro.2026.100786 (PMC13316230; doi:10.1016/j.ejro.2026.100786)
Supplement: Supplementary file 1 — Supplementary material [file mmc1.docx]

**Supplementary Appendix A:** Search strategy for each database

| Pubmed | ("Radiomics"[Mesh] OR Radiomics[all fields] OR Texture[all fields] OR "Deep Learning"[Mesh] OR "Machine Learning"[Mesh] OR "Deep learning"[all fields] OR "Machine learning"[all fields] OR CNN[all fields] OR "Neural Network"[all fields]")  AND  ("Magnetic Resonance Imaging"[Mesh] OR MRI OR "magnetic resonance imaging" OR "functional MRI" OR "diffusion-weighted imaging" OR "DCE-MRI" OR "multiparametric MRI")  AND  ("Neoadjuvant Therapy"[Mesh] OR "neoadjuvant chemotherapy" OR "induction chemotherapy" OR "preoperative chemotherapy" OR "Response" OR pCR OR " histopathological remission" OR "ypT0N0")  AND  ("Head and Neck Neoplasms"[Mesh] OR "head and neck cancer" OR HNSCC OR "oral cavity cancer" OR "oropharyngeal cancer" OR "laryngeal carcinoma" OR "nasopharyngeal carcinoma" OR "hypopharyngeal cancer") |
| --- | --- |
| Embase | ('radiomics'/exp OR radiomics.ti,ab,kw. OR texture.ti,ab,kw. OR 'deep learning'/exp OR 'machine learning'/exp OR 'deep learning'.ti,ab,kw. OR 'machine learning'.ti,ab,kw. OR cnn.ti,ab,kw. OR 'neural network'.ti,ab,kw.)  AND  ('magnetic resonance imaging'/exp OR 'MRI' OR 'functional MRI' OR 'diffusion weighted imaging' OR 'DCE MRI' OR 'multiparametric MRI')  AND  ('neoadjuvant chemotherapy'/exp OR 'induction chemotherapy' OR 'preoperative chemotherapy' OR 'response' OR 'histopathological remission' OR 'ypT0N0')  AND  ('head and neck cancer'/exp OR 'head and neck squamous cell carcinoma' OR 'oral cavity cancer' OR 'oropharyngeal cancer' OR 'nasopharyngeal carcinoma' OR 'laryngeal carcinoma' OR 'hypopharyngeal cancer') |
| Scopous | TITLE-ABS-KEY(radiomics OR "Deep learning" OR "Machine learning" OR CNN OR "Neural Network")  AND  TITLE-ABS-KEY("magnetic resonance imaging" OR MRI OR "functional MRI" OR "DWI" OR "DCE-MRI" OR "multiparametric MRI")  AND  TITLE-ABS-KEY("neoadjuvant chemotherapy" OR "induction chemotherapy" OR "preoperative chemotherapy" OR "response" OR pCR OR " pathological remission" OR "ypT0N0")  AND  TITLE-ABS-KEY("head and neck cancer" OR HNSCC OR "oral cavity cancer" OR "oropharyngeal carcinoma" OR "nasopharyngeal carcinoma" OR "laryngeal carcinoma" OR "hypopharyngeal cancer") |
| Web of science | 1: TI=("radiomics" OR radiomics OR texture OR "deep learning" OR "machine learning" OR cnn OR "neural network*")  AND  TI=("magnetic resonance imaging" OR MRI OR "functional MRI" OR "diffusion-weighted imaging" OR "DCE-MRI" OR "multiparametric MRI")  AND  TI=("neoadjuvant chemotherapy" OR "induction chemotherapy" OR "preoperative chemotherapy" OR "response" OR pCR OR "pathological remission" OR "ypT0N0")  AND  TI=("head and neck cancer" OR HNSCC OR "oral cavity cancer" OR "oropharyngeal cancer" OR "nasopharyngeal carcinoma" OR "laryngeal carcinoma" OR "hypopharyngeal cancer").  2: AB=("radiomics" OR radiomics OR texture OR imaging OR "deep learning" OR "machine learning" OR cnn OR "neural network*")  AND  AB=("magnetic resonance imaging" OR MRI OR "functional MRI" OR "diffusion-weighted imaging" OR "DCE-MRI" OR "multiparametric MRI")  AND  AB=("neoadjuvant chemotherapy" OR "induction chemotherapy" OR "preoperative chemotherapy" OR "response" OR pCR OR "pathological remission" OR "ypT0N0")  AND  AB=("head and neck cancer" OR HNSCC OR "oral cavity cancer" OR "oropharyngeal cancer" OR "nasopharyngeal carcinoma" OR "laryngeal carcinoma" OR "hypopharyngeal cancer"). |

**Supplementary Appendix B:** Radiomics pipeline in included studies.

| **First Author et al. (year)** | **Year** | **Segmentation method** | **Segmentation tool** | **Feature selection (reduction) method** | **Number of features selected** | **Cross-validation method** |
| --- | --- | --- | --- | --- | --- | --- |
| Hu C et al. (2021) | 2021 | Manual | ITK-SNAP (v.3.4.0) | mRMR,LASSO | T2WI:7,T1WI:6 | LASSO model tuning used 10-fold cross-validation |
| Huang L et al. (2023) | 2023 | Manual | ITK-SNAP (v 3.6.0) | Intra-class correlation coefficient (ICC > 0.7),Mann–Whitney U test (P<0.05),Univariate logistic regression,Maximum relevance minimum redundancy (mRMR) | 8 features (T2WI_FS-based: GLCM, GLRLM, First-order features, Entropy, Contrast, etc.) | not mentioned |
| Yongfeng P et al. (2021) | 2021 | Manual | ITK-SNAP (v3.8.0) | ANOVA / Mann–Whitney U test Correlation analysis (Spearman, threshold = 0.9) LASSO regression | 2 ClusterShade_angle135_offset4 Correlation_AllDirection_offshel_SD | not mentioned |
| Wang Y et al. (2024) | 2024 | Manual | 3D Slicer(v 4.21) | (ICC) for reproducibility Mann-Whitney U test for outcome-related feature selection Spearman’s rank correlation for correlation analysis Greedy recursive elimination strategy LASSO regression for final feature selection | 8 features:5 radiomics,3pathomics | 10-fold cross-validation on the training set. For PSO-SVM, particle swarm optimization was used to optimize parameters. |
| Pan M et al. (2025) | 2025 | Manual | ITK-SNAP (v 3.8.0) | mRMR,LASSO | 7 features:CE-T1WI, 10 features:T2-FS | 10-fold cross-validation Used in feature selection and model evaluation |
| Bologna M et al. (2022) | 2022 | Manual | Philips Intellispace | Principal Component Analysis (PCA) | 3 principal components used for classification Chosen to maintain a high instances-to-features ratio (10–15) | 100 iterations of train-test split  Each split used 70% training / 30% testing |
| Yuan J et al. (2024) | 2024 | Manual | ITK-SNAP | Intraclass Correlation Coefficient (ICC > 0.8) t-test (p < 0.05) Pearson correlation (r > 0.9) mRMR LASSO | 32 features (after reduction and LASSO) | 10-fold cross-validation (for LASSO) 5-fold cross-validation (for hyperparameter tuning in model building) |
| Wei H et al. (2025) | 2025 | Manual | ITK-SNAP(v 2.2.0) | intraclass correlation coefficient [ICC] ≥ 0.8 t-tests, Mann-Whitney U tests, and Pearson correlation coefficients (PCC) | 8 features (Pre-NCIT:1 feature,Post-NCIT:2 features,Delta_model:2 features,Combined_model:3 features ) | not mentioned |
| Wang G et al. (2018) | 2018 | Manual | Picture Archiving and Communication System (PACS) | LASSO,logistic regression | T1WI-CE:5 features, combined(T1WI, T2WI, T1WI-CE , T2WI-FS ):16 features | Bootstrap validation (1000 samples for internal validation) |
| Guo Y et al. (2023) | 2023 | Manual | ITK-SNAP( v 3.4.0) | LASSO | 5 features | 10 times 10-fold cross-validation |
| Liu J et al. (2016) | 2016 | Manual | In-house program module developed for the study in MATLAB | Concordance correlation coefficient (CCC),Dynamic range (DR),Fisher coefficient | 52 features (T1: 15,T2:18,DWI:19) | Stratified 10-fold cross-validation for internal validation; |
| Wang A et al. (2023) | 2023 | Manual | ITK-SNAP (v 3.8.0) | mRMR,LASSO,Backward stepwise selection | 193 features (T2WI:101, T1WI-CE:92) | 10-fold cross-validation via minimum criteria |
| Xu H et al. (2022) | 2022 | Manual | ITK-SNAP (v 3.8.0) | Mann-Whitney U test,Spearman correlation analysis,mRMR,LASSO | 12 features selected from the combined CE-T1WI and T2WI dataset after LASSO analysis | 10-fold cross-validation used in LASSO analysis |
| Liao H et al. (2022) | 2022 | Manual | ITK-SNAP (v 3.8.0) | Interclass correlation coefficient (ICC) Student’s t-test or Mann–Whitney U-test and LASSO for logistic regression. | 24 features | 10 fold cross-validation |
| Liao H et al. (2025) | 2025 | Manual | ITK-SNAP | The ComBat method for multicenter harmonization, followed by interclass correlation coefficient filtering and statistical tests (Student’s t-test or Mann–Whitney U-test) | 15 LANPC tumor response-related radiomics features | not mentioned |
| Wang Y et al. (2025) | 2025 | Manual | 3D-Slicer | Spearman correlation tests (to identify linear relationships between features), followed by the random forest algorithm to rank the importance of features | 15 features from T_Radiomics, 9 features from N_Radiomics, and various features from deep learning models (Resnet18, Resnet34, etc.) were selected | 10-fold cross-validation |
| Wang Y et al. (2022) | 2022 | Manual | MIM Software Inc. (for tumor delineation) | Stepwise forward selection method | 25 features | Bootstrapping (1000 samples) for internal cross-validation |
| Qiu L et al. (2025) | 2025 | Manual | ITK-SNAP | using Pearson’s correlation coefficient and LASSO regression to reduce redundancy and ensure statistical significance | 27 radiomic features | 10-fold cross-validation |
| Li Z et al. (2023) | 2023 | Manual | ITK-SNAP(v 3.6.0) | ANOVA (Analysis of Variance) ,recursive feature elimination (RFE) | 4 features | 5-fold cross-validation |
| Zhu Y et al. (2024) | 2024 | Manual | ITK-SNAP | LASSO,Z scores method | (18 features) 10 features for the habitat3mm model,8 features for the habitat1mm model | 10-fold cross-validation |
| Chen Z et al. (2024) | 2024 | Manual | 3D-Slicer(v 4.13.0) | Z-score standardization,Mann-Whitney U test or Student’s t-test,Pearson correlation to remove highly correlated features (\|r\| > 0.80),LASSO | 14 features | 10-fold cross-validation |

**Supplementary Appendix C:** Comprehensive METRICS evaluation

**
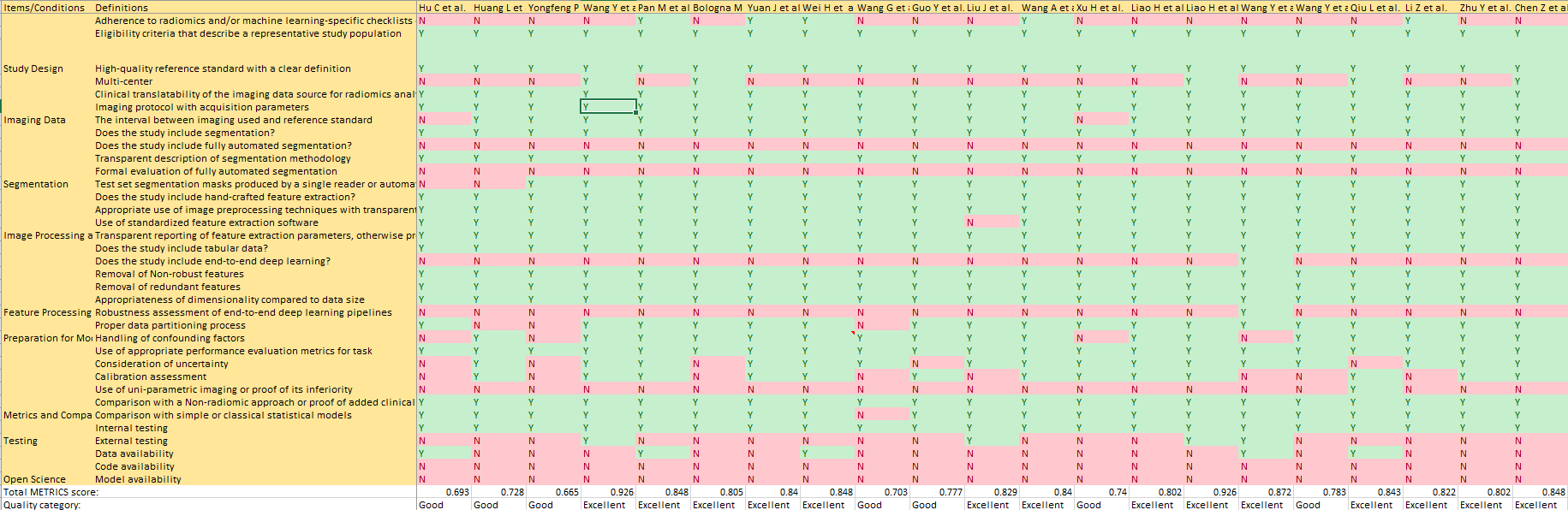
**

**Supplementary Appendix D:** Sensitivity analysis of studies distinguishing responders (CR/PR) from non-responders (PD/SD) among validation cohorts of radiomics-only models.

Sensitivity analysis of studies distinguishing responders (CR) from non-responders (PR/PD/SD) among validation cohorts of radiomics-only models

**Supplementary Appendix E:** Publication bias of studies distinguishing responders (CR/PR) from non-responders (PD/SD) among validation cohorts of radiomics-only models.

Publication bias of studies distinguishing responders (CR) from non-responders (PR/PD/SD) among validation cohorts of radiomics-only models.
